# Two New Species of *Fibrodontia* (*Trechisporales*, *Basidiomycota*) with a Key to Worldwide Species

**DOI:** 10.3390/jof7110982

**Published:** 2021-11-18

**Authors:** Shi-Liang Liu, Shuang-Hui He, Dong-Mei Liu, Li-Wei Zhou

**Affiliations:** 1 State Key Laboratory of Mycology, Institute of Microbiology, Chinese Academy of Sciences, Beijing 100101, China; liushiliang@im.ac.cn; 2 School of Ecology and Nature Conservation, Beijing Forestry University, Beijing 100083, China; heshuanghui@bjfu.edu.cn; 3 The Institute of Ecology, Chinese Research Academy of Environmental Sciences, Beijing 100012, China; ldmgenetics@163.com

**Keywords:** 2 new taxa, corticioid fungi, *Cystidiodendron*, *Hydnodontaceae*, taxonomy, wood-inhabiting fungi

## Abstract

*Fibrodontia* is a genus of wood-inhabiting fungi consisting of four species so far, including *F. gossypina* as generic type. Two new species, *Fibrodontia austrosinensis* and *F. subalba*, are described and illustrated from China. *Fibrodontia austrosinensis* from southwestern China is characterized by a grandinioid to odontioid hymenophore with numerous small aculei, a dimitic hyphal system with scattered, smooth skeletal hyphae and ellipsoid basidiospores measuring 4.2–5.2 × 3.5–4.5 μm. *Fibrodontia subalba* from the West Tianshan Mountain in northwestern China is distinguished by an odontioid to hydnoid hymenophore, a dimitic hyphal system, and ellipsoid basidiospores measuring 3.7–4.4 × 2.8–3.4 μm. The phylogenies inferred from the data set of nuc rDNA ITS1-5.8S-ITS2 (ITS) and D1–D2 domains of nuc 28S rDNA (28S), and that of ITS, 28S, translation elongation factor (*tef1α*), and RNA polymerase II second largest subunit (*rpb2*) supported *Fibrodontia* as a monophyletic genus in the *Trechisporales,* and *F. austrosinensis* and *F. subalba* as separate lineages within *Fibrodontia*. Multi-rate Poisson Tree Processes, Automatic Barcode Gap Discovery and genetic distance methods based on ITS sequences of *Fibrodontia* also supported *F. austrosinensis* and *F. subalba* as distinct species. The taxonomic status of *F. fimbriata* that was recently transferred from *Cystidiodendron*, is briefly discussed. A key to all six known species of *Fibrodontia* is provided.

## 1. Introduction

*Fibrodontia* Parmasto, introduced for *F. gossypina* Parmasto [1], resembles *Hyphodontia* J. Erikss. by its resupinate, light-colored basidiocarps with odontioid hymenophores, clamped generative hyphae, and hyaline, thin-walled basidiospores. However, the presence of skeletal hyphae and the absence of cystidia distinguish *Fibrodontia* from *Hyphodontia* [2]. Eriksson et al. believed that *Fibrodontia* was closely allied to *Hyphodontia* [2] and Langer considered it a synonym of *Hyphodontia* [3]. Surprisingly, molecular phylogenetic studies place these genera in different orders—*Fibrodontia* in the trechisporoid clade in the *Hydnodontaceae* Jülich, *Trechisporales* K.H. Larss. [4,5], and *Hyphodontia* in the *Hyphodontiaceae* Xue W. Wang & L.W. Zhou, *Hymenochaetales* Oberw. [6].

Besides *Fibrodontia gossypina*, the generic type, the genus includes *Fibrodontia brevidens* (Pat.) Hjortstam & Ryvarden, *F. tomentosa* (Berk. & M.A. Curtis) Hjortstam & Ryvarden, and *F. alba* Yurchenko & Sheng H. Wu [7,8]. Of the four species currently accepted in *Fibrodontia*, *F. alba* and *F. brevidens* are reported from China [8,9,10].

During a study of wood-inhabiting fungi in China, two undescribed species of *Fibrodontia* were identified based on morphological criteria and molecular genetic analyses. The primary purpose of this study is to use an integrative taxonomic approach for the delimitation and description of two new species of *Fibrodontia* from China.

## 2. Materials and Methods

### 2.1. Morphological Examination

The studied specimens, deposited in the fungarium of Institute of Microbiology, Chinese Academy of Sciences (HMAS), and the herbarium of Beijing Forestry University (BJFC), were macromorphologically observed with the aid of a Leica M 125 stereomicroscope (Wetzlar, Germany) at magnifications up to 100×. Special color terms followed Kornerup and Wanscher [11]. The microscopic procedure followed Wang et al. [12]. Specimen sections were mounted in Cotton Blue (CB, 0.1 mg aniline blue dissolved in 60 g pure lactic acid), Melzer’s reagent (1.5 g potassium iodide, 0.5 g crystalline iodine and 22 g chloral hydrate dissolved in 20 mL distilled water) or 5% potassium hydroxide (KOH), then examined with an Olympus BX43 light microscope (Tokyo, Japan) at magnifications up to 1000×. All measurements were taken from the sections mounted in CB. When reporting the variation in the size of the basidiospores, 5% of measurements were excluded from each end of the range and are given in parentheses. L stands for mean basidiospore length (arithmetic average of all basidiospores), W for basidiospore width (arithmetic average of all basidiospores), Q for variation in the ratio of L to W among the studied specimens, and n (a/b) for number of basidiospores (a) measured from given number of specimens (b). Drawings were made with the aid of a drawing tube.

### 2.2. DNA Amplification and Sequencing

Total DNA was extracted from selected specimens using the CTAB rapid plant genome extraction kit (Aidlab Biotechnologies Co., Ltd., Beijing, China) according to the manufacturer’s instructions. The nuc rDNA ITS1-5.8S-ITS2 (ITS) region was amplified with primers ITS5 and ITS4 [13], the D1–D2 domains of nuc 28S rDNA (28S) with primers LR0R and LR7 [14], the translation elongation factor (*tef1α*) with primers 983F and 1567R [15], and the RNA polymerase II second largest subunit (*rpb2*) with primers RPB2-f5F and RPB2-b7.1R [16,17] using 2×EasyTaq^®^ PCR SuperMix (TransGen Biotech Co., Ltd., Beijing, China). The PCR procedure was as follows: for ITS and *tef1α*, initial denaturation at 95 °C for 3 min, followed by 35 cycles at 94 °C for 40 s, 54 °C for 45 s and 72 °C for 1 min, and a final extension of 72 °C for 10 min; for 28S, initial denaturation at 94 °C for 1 min, followed by 34 cycles at 94 °C for 30 s, 50 °C for 1 min, 72 °C for 1.5 min, and a final extension of 72 °C for 10 min; for *rpb2*, initial denaturation at 94 °C for 2 min, followed by 10 cycles at 94 °C for 45 s, 60 °C for 45 s (minus 1 °C per cycle) and 72 °C for 1.5 min, then followed by 36 cycles at 94 °C for 45 s, 53 °C for 1 min and 72 °C for 1.5 min, and a final extension of 72 °C for 10 min. The PCR product was sequenced with the same primers used in PCR amplification at the Beijing Genomics Institute, China. The newly generated sequences were submitted to GenBank (http://www.ncbi.nlm.nih.gov/genbank; Table 1).

### 2.3. Phylogenetic Analyses

To explore the phylogenetic position of the newly sequenced Chinese specimens, a combined data set of ITS and 28S region adapted from the data sets used in Yurchenko and Wu [8] and Liu et al. [10] was employed. This updated data set included all main lineages in *Trechisporales* as ingroup taxa with *Hyphodontia subalutacea* (P. Karst.) J. Erikss. and *H. floccosa* (Bourdot & Galzin) J. Erikss. from *Hymenochaetales* as outgroup taxa. A second data set with a combination of ITS, 28S, *tef1α* and *rpb2* region was used to further explore the relationships of the newly sequenced Chinese specimens within *Fibrodontia*. Besides species of *Fibrodontia*, other related taxa in *Hydnodontaceae* with at least one of *tef1α* and *rpb2* region available were included. An unnamed species of *Sistotremastrum* J. Erikss. with a separate family position from *Hydnodontaceae* in *Trechisporales* [5] was selected as the outgroup taxon. A third data set with the ITS region of *Fibrodontia* was used to confirm the species independency.

Of the three data sets, each gene region was separately aligned using MAFFT 7.110 [18] with the G-INS-i strategy [19] and then concatenated to three alignments accordingly. The resulting alignments were deposited at TreeBase (http://www.treebase.org; submission ID S27597). The best-fit evolutionary model for each gene region of the three alignments was separately estimated using jModelTest 2.1.10 [20,21]. Following these models, Bayesian inference (BI) and maximum likelihood (ML) algorithms were used to performed phylogenetic analysis. BI was conducted using MrBayes 3.2.7a [22]. Two independent runs were employed. Each run started from random trees and had 1,000,000 generations. Trees were sampled every 1000th generation. The first 25% of sampled trees were discarded as burn-in, whereas all remaining trees were used to construct a 50% majority consensus tree and for calculating Bayesian posterior probabilities (BPPs). Chain convergence was determined using Tracer 1.7 [23]. ML analysis was performed using raxmlGUI 2.0 [24,25] and bootstrap (BS) replicates were evaluated under the auto FC option [26].

Regarding the tree inferred from the data set of ITS region, species delimitation was further estimated using multi-rate Poisson Tree Processes (mPTP) [27] with default parameters and Automatic Barcode Gap Discovery (ABGD) [28] with P_min_ = 0.01, P_max_ = 0.1, steps = 20, X (relative gap width) = 1.5, Nb bins (for distance distribution) = 20 and Jukes-Cantor model (JC69). In addition, interspecific and intraspecific genetic distances of ITS sequences were calculated from the alignment of the data set of ITS region using MEGA X [29] with a bootstrap method of variance estimation in 1000 BS replications, a *p*-distance substitution model, the uniform rates among sites, and a pairwise deletion treatment.

## 3. Results

**Figure 1:**
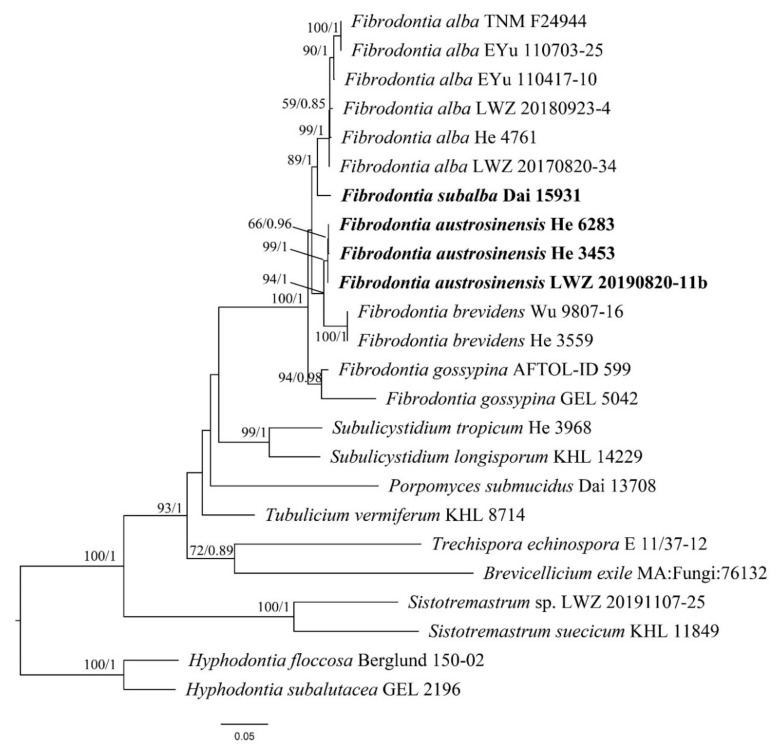
Phylogenetic position of *Fibrodontia austrosinensis* and *F. subalba* inferred from the data set of ITS and 28S region. The topology is from maximum likelihood analysis. Bootstrap values and Bayesian posterior probabilities, if simultaneously above 50% and 0.8, respectively, are labelled at the nodes. The newly described species are in boldface.

**Figure 2:**
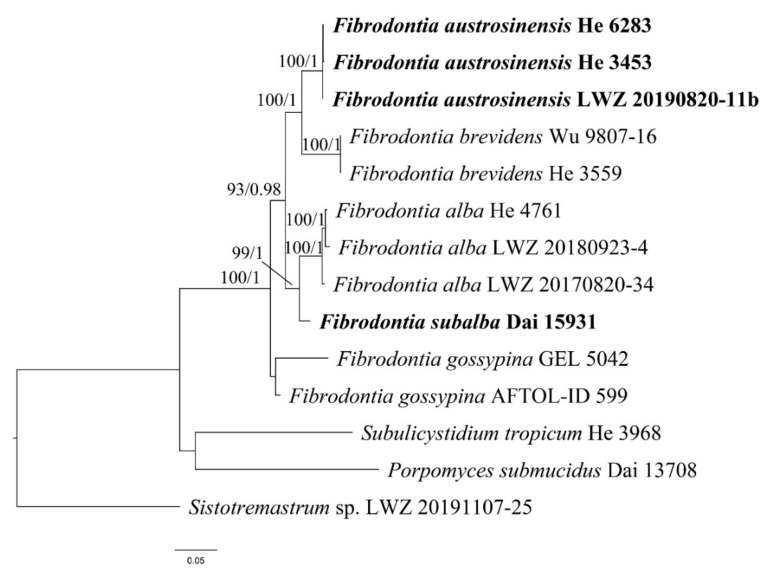
Phylogenetic position of *Fibrodontia austrosinensis* and *F. subalba* inferred from the data set of ITS, 28S, *tef1α*, and *rpb2* region. The topology is from maximum likelihood analysis. Bootstrap values and Bayesian posterior probabilities, if simultaneously above 50% and 0.8, respectively, are labelled at the nodes. The newly described species are in boldface.

**Figure 3:**
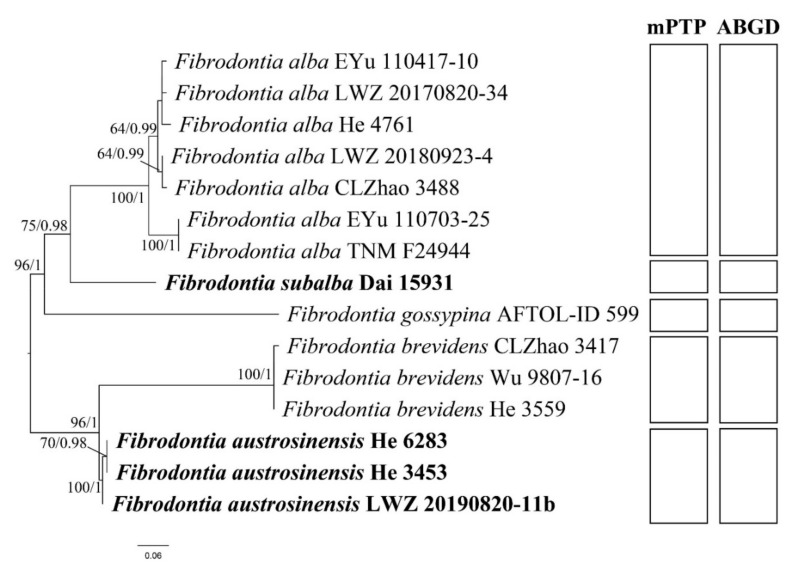
Phylogenetic relationship of species within *Fibrodontia* inferred from the data set of ITS region. The topology is from maximum likelihood analysis. Bootstrap values and Bayesian posterior probabilities, if simultaneously above 50% and 0.8, respectively, are labelled at the nodes. The newly described species are in boldface. Species delimitation indicated by rectangles was inferred using the multi-rate Poisson Tree Processes (mPTP) and the Automatic Barcode Gap Discovery (ABGD) methods.

The data set of ITS and 28S region included 22 ITS and 21 28S sequences from 24 samples, and resulted in an alignment of 2011 characters. The data set of ITS, 28S, *tef1α* and *rpb2* region included 12 ITS, 12 28S, eight *tef1α* and nine *rpb2* sequences from 14 samples, and resulted in an alignment of 3115 characters. The data set of ITS region with 15 samples and resulted in an alignment of 593 characters. The ML searches for the data sets of ITS and 28S region, ITS, 28S, *tef1α* and *rpb2* region and ITS region stopped after 200, 100 and 350 replicates, respectively. For BI, GTR + I + G was estimated as the best-fit evolutionary model for the data sets of ITS and 28S region and ITS, 28S, *tef1α* and *rpb2* region, while HKY + I as that for data set of ITS region. For the three data sets, all chains in BI converged after 1,000,000 generations, where the effective sample sizes of all parameters were greater than 400 and the potential scale reduction factors approached 1.000. Because BI and ML algorithms generated nearly congruent topologies, the ML trees are presented with BS values and BPPs greater than 50% and 0.8, respectively, shown at the nodes (Figure 1, Figure 2 and Figure 3).

The phylogeny inferred from the data set of ITS and 28S region (Figure 1) recovered *Fibrodontia* as a monophyletic genus within *Trechisporales*. The phylogenies inferred from the data sets of ITS and 28S region (Figure 1) and ITS, 28S, *tef1α* and *rpb2* region (Figure 2) both recovered *F. alba*, *F. brevidens* and *F. gossypina* as distinct lineages. In addition, Dai 15,931 from northwestern China represented a distinct lineage sister to *F. alba* (BS = 89%, BPP = 1.00 in Figure 1; BS = 99%, BPP = 1.00 in Figure 2) and three samples from southwestern China, viz. He 3453, He 6283, LWZ 20190820-11b, formed a strongly supported lineage sister to *F. brevidens* (BS = 94%, BPP = 1.00 in Figure 1; BS = 100%, BPP = 1.00 in Figure 2). The midpoint-rooted phylogenetic tree inferred from the data set of ITS region strongly supported the independence of each species, which strictly corresponded to the species delimitation inferred using mPTP and ABGD methods (Figure 3). Moreover, the mean values of genetic distances calculated from the data set of ITS region between the species of *Fibrodontia* ranged from 5.54% to 10.45%, compared to intraspecific distances of 0.06% to 1.17% (Table 2). Taking morphological characters into consideration, these two new lineages represent new species that are described below.

## 4. Taxonomy

**Figure 4:**
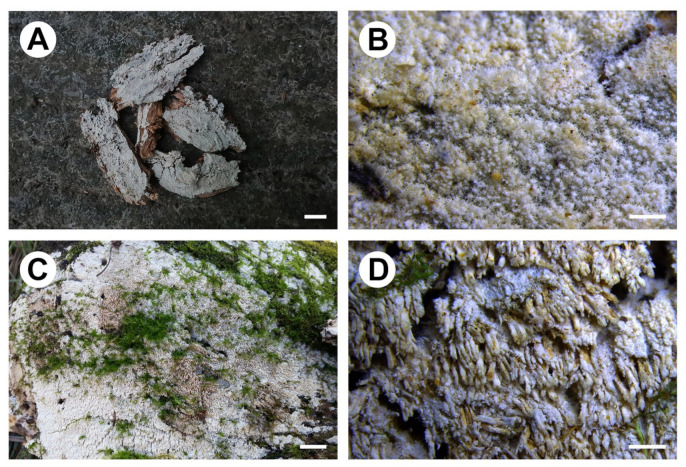
Basidiocarps of *Fibrodontia*. (**A**,**B**) *F. austrosinensis* (holotype); (**C**,**D**) *F. subalba* (holotype).—Scale bars: (**A**,**C**) = 1 cm; (**B**) = 0.5 mm; (**D**) = 1 mm.

**Figure 5:**
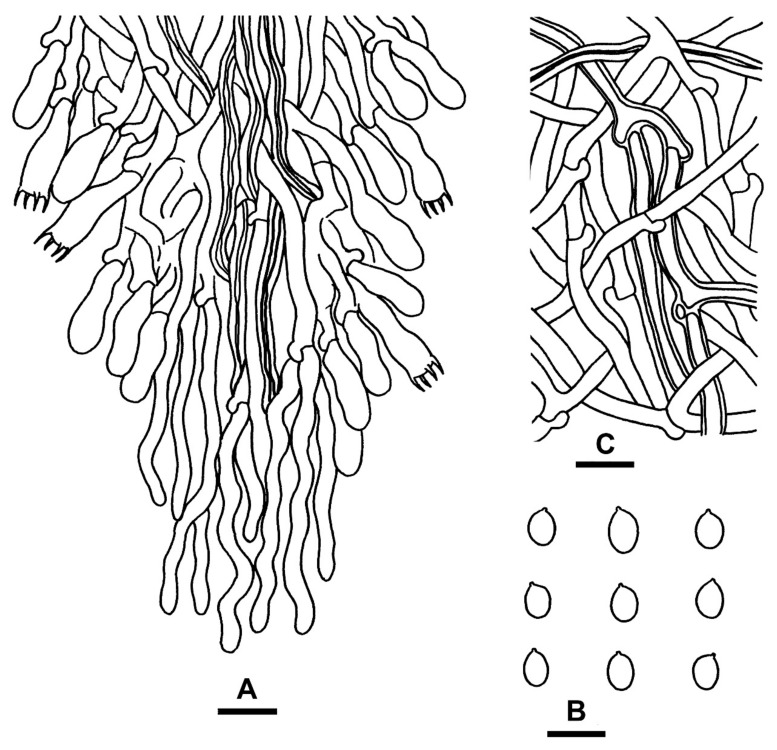
Microscopic structures of *Fibrodontia austrosinensis* (drawn from the holotype). (**A**) Part of the vertical section of basidiocarp; (**B**) basidiospores; (**C**) hyphae from subiculum. Scale bars: (**A**,**B**,**C**) = 10 µm.

***Fibrodontia austrosinensis*** S.L. Liu, S.H. He & L.W. Zhou, **sp. nov.** (Figure 4A,B and Figure 5)

**MycoBank: MB 836338.**

**Etymology:***austrosinensis* (Latin), refers to the distribution in southern China.

**Type:** China, Sichuan, Pingshan County, Laojunshan National Nature Reserve, 104°4′ E, 28°40′ N, on rotten wood of angiosperm, 20 August 2019, *L.W. Zhou*, LWZ 20190820-11b (holotype HMAS).

**Description:** Basidiocarps annual, resupinate, inseparable from substrate, without odor or taste, soft corky, and brittle when dry, up to 8 cm long, 4 cm wide and 0.5 mm thick. Hymenophore grandinioid to odontioid with numerous small aculei, darkening but otherwise unchanged in KOH. Aculei orange white (5A2), greyish-orange (5B6) to yellowish brown (5D5) when fresh, greyish orange (5B3) to brownish orange [5C(3–4)] when dry, apically slightly fimbriate, up to 0.4 mm long. Subiculum white to cream, up to 100 μm thick. Margin white, cottony, up to 0.5 mm wide. Hyphal system dimitic; generative hyphae with clamp connections. Subiculum composed of a loose layer of distinct hyphae; generative hyphae, hyaline, thin- to slightly thick-walled, occasionally branched, smooth, 2–3.5 μm in diam; skeletal hyphae rare, hyaline to yellowish, thick-walled with a wide to narrow lumen, unbranched, smooth, slightly flexuous, loosely interwoven, 2–3 μm in diam. Aculei composed of a central core of compact hyphae and subhymenial and hymenial layers, at apex terminal hyphae slightly tapered; generative hyphae distinct, hyaline, thin- to slightly thick-walled, occasionally branched, smooth, interwoven, 2–3 μm in diam; skeletal hyphae rare, hyaline to yellowish, thick-walled with a wide to narrow lumen, unbranched, smooth, slightly flexuous, more or less parallel along the aculei, 2–3 μm in diam. Basidia suburniform to clavate, thin-walled, with four sterigmata and a clamp connection at base, 13–16 × 4.5–5.5 μm; basidioles similar in shape to basidia, but smaller. Basidiospores ellipsoid to ovoid, hyaline, thin-walled, smooth, inamyloid, indextrinoid, acyanophilous, (4–)4.2–5.2(–5.5) × (3–)3.5–4.5(–4.8) μm, L = 4.8 μm, W = 3.9 μm, Q = 1.2–1.3 (*n* = 90/3).

Other specimens (paratypes) examined: China, Yunnan, Tengchong County, Laifengshan Forest Park, 98°29′ E, 25°1′ N, on rotten wood of angiosperm, 1 Dec. 2015, *S.H. He*, He 3453 (BJFC); Xichou County, Xiaoqiaogou Forest Park, 104°41′ E, 23°21′ N, on rotten wood of angiosperm, 16 Nov. 2019, *S.H. He*, He 6283 (BJFC).

**Notes:***Fibrodontia austrosinensis* resembles species of *Hyphodontia* s.l., since it has grandinioid to odontioid hymenophore with numerous, short aculei and ellipsoid basidiospores. However, development of skeletal hyphae in the subiculum and aculei trama places this taxon in *Fibrodontia*. *Fibrodontia brevidens* resembles *F. austrosinensis* but differs in producing slightly longer aculei, up to 0.7 mm long, moderately encrusted skeletal hyphae, and shorter, subglobose basidiospores (4–4.5 × 3.5–4.5 μm) [3].

**Figure 6:**
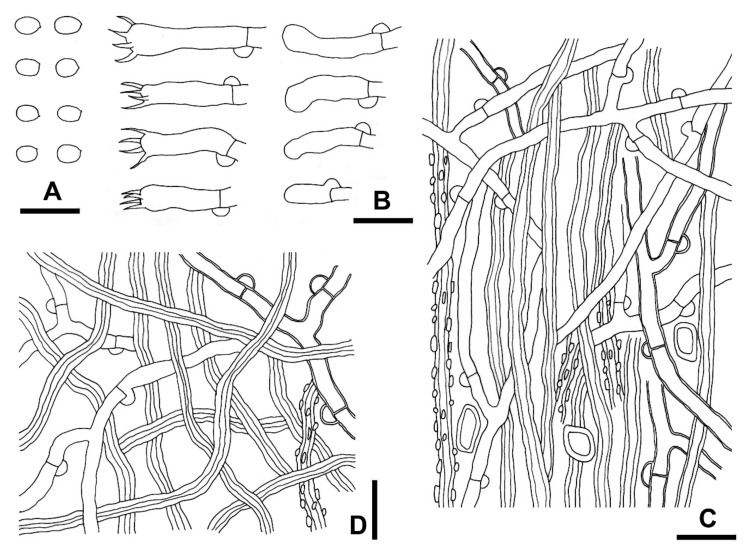
Microscopic structures of *Fibrodontia subalba* (drawn from the holotype). (**A**) Basidiospores; (**B**) basidia and basidioles; (**C**) hyphae from trama; (**D**) hyphae from subiculum. Scale bars: (**A**,**B**,**C**,**D**) = 10 µm.

***Fibrodontia subalba*** S.L. Liu & L.W. Zhou, **sp. nov.** (Figure 4C,D and Figure 6)

**MycoBank: MB 836339.**

**Etymology:***subalba* (Latin), refers to the similarity to *Fibrodontia alba*.

**Type:** China, Xinjiang, Gongliu County, West Tianshan Mountain National Nature Reserve, 82°49′ E, 43°1′ N, on rotten wood of *Populus*, 14 Sept. 2015, *Y.C. Dai*, Dai 15,931 (holotype BJFC).

**Description:** Basidiocarps annual, resupinate, inseparable from substrate, without odor or taste, soft corky, and brittle when dry, up to 15 cm long, 10 cm wide and 1.7 mm thick. Hymenophore odontioid to hydnoid, darkening but otherwise unchanged in KOH. Aculei orange white (5A2) when fresh, turning yellowish brown [5D(4–6)] with age, greyish yellow [4B(3–4)] dry, 3–5 per mm, apically slightly fimbriate, up to 1.5 mm long. Subiculum white to cream, up to 0.2 mm thick. Margin white, cottony, up to 0.7 mm wide. Hyphal system dimitic; generative hyphae with clamp connections. Subiculum composed of a loose layer of distinct hyphae; generative hyphae, hyaline, thin- to slightly thick-walled, occasionally branched, smooth, 2–3.5 μm in diam; skeletal hyphae hyaline to yellowish, thick-walled with a wide to narrow lumen, unbranched, smooth or rarely encrusted with faceted crystals, more or less flexuous, loosely interwoven, 1.5–3 μm in diam. Aculei composed of a central core of compact hyphae and subhymenial and hymenial layers, at apex terminal hyphae slightly tapered; generative hyphae hyaline, thin- to slightly thick-walled, occasionally branched, smooth, interwoven, 2–4 μm in diam; skeletal hyphae hyaline to yellowish, thick-walled with a wide lumen, unbranched, smooth or occasionally encrusted with faceted crystals, mainly straight, more or less parallel along the aculei, 1.5–3.5 μm in diam. Basidia suburniform to clavate, thin-walled, with four sterigmata and a clamp connection at base, 12–20 × 4–6 μm; basidioles similar in shape to basidia, but smaller. Large rhomboid crystals present in subiculum and aculei. Basidiospores ellipsoid, hyaline, thin-walled, smooth, inamyloid, indextrinoid, acyanophilous, (3.5–)3.7–4.4(–4.6) × (2.7–)2.8–3.4(–3.5) μm, L = 4.0 μm, W = 3.1 μm, Q = 1.3 (*n* = 30/1).

**Notes:***Fibrodontia subalba* is characterized by resupinate, odontioid to hydnoid basidiocarps with long aculei, a dimitic hyphal system with clamped generative hyphae and skeletal hyphae, absence of cystidia, and ellipsoid, hyaline, thin-walled, smooth basidiospores. *Fibrodontia subalba* is quite similar to *F. alba* but the latter species differs by denser (6–10 per mm), shorter aculei (up to 0.4 mm long), and slightly narrower basidiospores (2.5–3.5 μm wide) [8]. Moreover, *F. alba* is reported from subtropical evergreen broad-leaved forests [8], whereas *F. subalba* is from a semiarid continental region. From other species of *Fibrodontia*, *F. subalba* is distinguished by its significantly longer aculei (up to 1.5 mm long).
**A key to worldwide species of *Fibrodontia***
1a. Hymenophore odontioid to hydnoid, aculei up to 1.5 mm long2b. Hymenophore grandinioid to odontioid, aculei up to 0.7 mm long32a. Aculei usually less than 1 mm long, skeletal hyphae (2.5–)3.5–4.5 μm wide*F. gossypina*b. Aculei usually more than 1 mm long, skeletal hyphae 1.5–3.5 μm wide*F. subalba*3a. Basidiospores generally 7–9 μm long*F. tomentosa*b. Basidiospores generally 3.5–5.5 μm long44a. Skeletal hyphae smooth*F. austrosinensis*b. Skeletal hyphae usually encrusted with crystals55a. Basidiospores generally broadly ellipsoid to globose, 4.5–5 × 3.5–4.5 μm*F. brevidens*b. Basidiospores generally ellipsoid to ovoid, 3.5–4.5 × 2.5–3 μm*F. alba*

## 5. Discussion

In this study, two species of *Fibrodontia* from China, *F. austrosinensis* and *F. subalba*, are described as new species based on molecular analyses and morphological features. Phylogenetic analyses on five species of *Fibrodontia* presented above confirmed that *Fibrodontia* is a monophyletic genus in a strongly supported clade (Figure 1). *Fibrodontia austrosinensis* and *F. subalba* were clearly separated from other sampled species of *Fibrodontia* and from each other (Figure 1, Figure 2 and Figure 3). Both mPTP and ABGD methods correspondingly confirmed the delimitation of the five sampled species of *Fibrodontia* (Figure 3). Moreover, based on ITS region (Table 2), intraspecific difference within specimens of *F. alba* was 1.17%, whereas interspecific difference between sister taxa *F. subalba* and *F. alba* was 5.54%. Similarly, interspecific sequence difference between sister taxa *F. brevidens* and *F. austrosinensis* was 5.30% compared to intraspecific differences of 0.06% and 0.12%, respectively (Table 2).

The type of *Fibrodontia brevidens* was originally collected in Ecuador [7] and this species is considered to be widely distributed [9,30]. Ideally the sequences generated from samples in or close to type localities are included in phylogenetic analyses. However, the sequences of *F. brevidens* from South American samples are unavailable. Instead, in this study we used the sequences generated from Asian samples to represent *F. brevidens* following Yurchenko and Wu [8]. Molecular evidence supported *F. brevidens* as a distinct species within *Fibrodontia* (Figure 1, Figure 2 and Figure 3; Table 2).

*Fibrodontia tomentosa* was absent from the current phylogeny. However, *F. tomentosa* is morphologically unique within this genus by its almost grandinioid hymenial surface, large rhomboid crystals present among hyphae, and cylindric to suballantoid basidiospores measured as 7–9 × 3.5–4.5 μm [3,8]. *Fibrodontia tomentosa* seems to be an uncommon species in tropical American countries and is so far known only from Cuba and Venezuela [3,7,31].

Baltazar et al. [32] studied the type of *Cystidiodendron fimbriatum* Rick and transferred it to *Fibrodontia* as *F. fimbriata* (Rick) Baltazar & Rajchenb. Furthermore, they proposed that *F. fimbriata* and *F. gossypina* are conspecific based on their morphological features. If this synonym was confirmed, the monotypic genus *Cystidiodendron* Rick and its generic type *C. fimbriatum*, both introduced by Rick [33], would have priority over *Fibrodontia* (published in 1968), and thus all six species of *Fibrodontia* should be transferred to the genus *Cystidiodendron*. To keep taxonomic status of *Fibrodontia* stable, however, we do not propose any taxonomic changes related to this issue until molecular sequences from *C. fimbriatum* are available for phylogenetic analyses.

## Figures and Tables

**Table 1:** Sequences used in the phylogenetic analyses.

Species	Voucher Number	GenBank Accession Number			
		ITS	28S	*tef1α*	*rpb2*
*Brevicellicium exile*	MA:Fungi:76132	HE963779	—	—	—
*Fibrodontia alba*	TNM F24944	NR153983	NG060401	—	—
	EYu 110703-25	KC928274	KC928275	—	—
	EYu 110417-10	JQ612713	JQ612714	—	—
	He 4761	MK204529	MK204541	**MW478697**	**MW478705**
	LWZ 20170820-34	**MT802108**	**MT802102**	**MW478698**	**MW478706**
	LWZ 20180923-4	**MT802107**	**MT802101**	**MW478696**	**MW478704**
	CLZhao 3488	MK268910	—	—	—
*Fibrodontia austrosinensis*	LWZ 20190820-11b	**MT802111**	**MT802105**	**MW478700**	**MW478709**
	He 3453	**MT802109**	**MT802103**	—	**MW478708**
	He 6283	**MT802110**	**MT802104**	**MW478699**	**MW478710**
*Fibrodontia brevidens*	Wu 9807-16	KC928276	KC928277	—	—
	He 3559	MK204528	—	**MW478701**	**MW478707**
	CLZhao 3417	MK268911	—	—	—
*Fibrodontia gossypina*	AFTOL-ID 599	DQ249274	AY646100	—	—
	GEL 5042	—	AJ406421	—	—
*Fibrodontia subalba*	Dai 15931	**MT802106**	**MT802100**	—	—
*Hyphodontia subalutacea*	GEL 2196	DQ340341	DQ340362	—	—
*Hyphodontia floccosa*	Berglund 150-02	DQ873618	DQ873618	—	—
*Porpomyces submucidus*	Dai 13708	KT152144	KT152146	**MW478702**	—
*Sistotremastrum suecicum*	KHL 11849	EU118666	EU118667	—	—
*Sistotremastrum* sp.	LWZ 20191107-25	**MW474864**	**MW477771**	**MW478703**	**MW478712**
*Subulicystidium longisporum*	KHL 14229	MH000601	MH000601	—	—
*Subulicystidium tropicum*	He 3968	MK204531	MK204544	—	**MW478711**
*Trechispora echinospora*	E 11/37-12	JX392853	JX392854	—	—
*Tubulicium vermiferum*	KHL 8714	—	AY463477	—	—

The newly generated sequences are in boldface.

**Table 2:** Genetic distances of ITS sequences between and within species of *Fibrodontia*.

	Species	1	2	3	4	5
1	*F. alba*	*0.0117*				
2	*F. subalba*	0.0554	*n.a.*			
3	*F. brevidens*	0.0719	0.0758	*0.0006*		
4	*F. austrosinensis*	0.0554	0.0592	0.0530	*0.0012*	
5	*F. gossypina*	0.0879	0.0903	0.1045	0.0781	*n.a.*

The mean values of genetic distances between species are shown below the diagonal, and those within species are shown in italic along the diagonal.

## Data Availability

Publicly available datasets were analyzed in this study. All resulting alignments were deposited in TreeBASE (http://www.treebase.org; accession number S27597). All newly generated sequences were deposited in GenBank (https://www.ncbi.nlm.nih.gov/genbank/; Table 1). All new taxa were deposited in MycoBank (https://www.mycobank.org/; MycoBank identifiers follow new taxa).
